# Vestibulocochlear toxicity in a pair of siblings 15 years apart secondary to aspartame: two case reports

**DOI:** 10.1186/1757-1626-0002-0000009237

**Published:** 2009-09-15

**Authors:** Paul Pisarik, Dasha Kai

**Affiliations:** 1 University of Oklahoma College of Medicine, Tulsa, 1111 S. St. Louis Ave. Tulsa, OK 74120-5440, USA; 2 University Physician's Hospital, 2800 E. Ajo Way, Tucson, AZ 85713, USA

## Abstract

**Introduction:**

Aspartame may have idiosyncratic toxic effects for some people; however, there are few case reports published in the medical literature. We present two case reports in a pair of siblings, one with a vestibular and the other with a cochlear toxicity to aspartame. The cochlear toxicity is the first case to be reported, while the vestibular toxicity is the second case to be reported.

**Case presentation:**

A 29-year-old white female had a 20-month history of nausea and headache, progressively getting worse with time and eventually to also involve vomiting, vertigo, and ataxia. She was extensively evaluated and diagnosed with a vestibular neuronitis versus a chronic labyrinthitis and treated symptomatically with limited success. In response to a newspaper article, she stopped her aspartame consumption with total cessation of her symptoms. Fifteen years later, her then 47-year-old white brother had a 30-month history of an intermittent, initially 5-10 minute long episode of a mild sensorineural hearing loss in his right ear that progressed over time to several hour episodes of a moderately severe high-frequency sensorineural hearing loss to include tinnitus and a hypoesthetic area in front of his right tragus. After a negative magnetic resonance scan of the brain, he remembered his sister's experience with aspartame and stopped his consumption of aspartame with resolution of his symptoms, although the very high frequency hearing loss took at least 15 months to resolve. For both, subsequent intentional challenges with aspartame and unintentional exposures brought back each of their respective symptoms.

**Conclusion:**

Aspartame had a vestibulocochlear toxicity in a pair of siblings, suggesting a genetic susceptibility to aspartame toxicity. Even though the yield may be low, asking patients with dizziness, vertigo, tinnitus, or high-frequency hearing loss about their aspartame consumption and suggesting cessation of its use, may prove helpful for some.

## Introduction

Aspartame was approved by the United States Food and Drug Administration (FDA) in 1981 for use in dry products such as breakfast cereal and as a tabletop sweetener [1]. Later in 1983 it was approved for use in sodas and in 1995 as a general sweetener in all foods and drinks. Because of its very sweet taste, aspartame has been extensively used as a food additive. It is included in over 6000 products and consumed by 200 million people around the world [2]. Because of its ubiquity in our food supply, the FDA has received many complaints over the years that allege that aspartame was responsible for a myriad of consumers' illnesses.

A PubMed search only shows only 14 case reports or case series regarding potential aspartame toxicity. The neurotoxic reactions reported include migraines, carpal tunnel syndrome, a movement disorder of the arms and legs, an orofacial sensitivity reaction, vertigo and ataxia, and seizure and mania. This will be the first paper to show a sensorineural hearing loss temporally related to aspartame and the second to show a vestibular toxicity temporally related to aspartame [3].

## Case presentation

### Case report 1

The first case was a 29-year-old white non-Hispanic female nurse (one of the authors) who started experiencing nausea and vomiting 3 weeks after conceiving her first pregnancy that persisted throughout her pregnancy. Her pregnancy was complicated with a 114-pound weight gain and pre-eclampsia. Her delivery was complicated with shoulder dystocia and post partum hemorrhage. After her delivery in January 1985, her symptoms got better in that she only had nausea in the mornings that cleared by 1 or 2 P.M. She also had headaches on awakening in the morning up to 3 days a week.

She was on no medications and had no allergies to medications. She denied use of alcohol, cigarettes, or any other drugs. Her past medical history was significant for intermittent hypertension. She had multiple right eye surgeries for strabismus in the early 1960s and a tonsillectomy and adenoidectomy in 1965. She was 5 foot and 11 inches tall and weighed 165 lbs prior to her pregnancy, 264 lbs just after her delivery that decreased over the time to 180 lbs at time of her self-diagnosis.

She went to see her physician in July 1985 with these symptoms. Her physician treated her with oral prochlorperazine and meclizine; however, these medications did not have much effect on her.

In January 1986, she again saw her physician with the same complaints. The neurological exam was normal except for showing a few beats of nystagmus on lateral gaze. She was again treated symptomatically with metoclopramide and trimethobenzamide. Over the next several months, the nausea progressed to encompass the entire day along with some vomiting. In addition, she was experiencing vertigo with motion and lying in bed.

She saw her physician in June 1986 and was noted to have nonfatiguing nystagmus on looking to the right. She had audiologic testing that was normal but had an electronystagmograph (ENG) that showed a direction-fixed right-beating positional nystagmus of about six degrees. She was referred to an otolaryngologist who noted a positioning nystagmus consistent with the ENG that was easily reproduced and persistent. Radiographs of the internal auditory canals were normal. Because of the persistence of her symptoms, she was then referred to a neurologist. He ordered a brainstem auditory evoked response test and a magnetic resonance imaging scan of the brain, both of which were normal. His final diagnosis was a vestibular neuronitis versus a chronic labyrinthitis and he gave her diazepam to take as needed.

Over the next two months, her symptoms got worse to where she was experiencing problems with muscle coordination manifested by occasionally not being able to negotiate doorways and occasionally not being able to place a spoon squarely in her mouth.

In September 1986, she read a doctor-advice column in the local paper that mentioned aspartame was anecdotally associated with nausea and headaches. She had first begun to drink an aspartame-sweetened drink - Crystal Light® - right after the birth of her son and was drinking 16 to 32 ounces per day, sipping on it throughout the day. She made sure that she did not consume it during her pregnancy. After reading the article, she stopped drinking Crystal Light®, her only source of aspartame, and within a week, she was symptom free. About a month later, she challenged herself with 8 ounces of Crystal Light® and within 1 to 2 hours started having nausea, headaches, and vertigo that lasted for 48 hours. About 3 to 4 weeks later she drank a 12-ounce can of Diet Pepsi® and within 1 hour started to have the exact same symptoms, again lasting about 48 hours. She had a recurrence of the symptoms two times after that, each time after accidentally drinking a beverage with aspartame in it. After the last occurrence of her nausea, headaches, and vertigo, she has since not consumed aspartame in any product or form and has been symptom free for 22 years.

Primary diagnosis is a vestibular neuronitis versus chronic labyrinthitis secondary to aspartame. Secondary diagnosis is nausea and vomiting of pregnancy.

### Case report 2

In January of 2002 at the age of 47, a non-Hispanic white male physician (one of the authors and brother of case report 1) had an intermittent right-sided tinnitus associated with a hearing loss that would last 6-8 hours at a time. In addition, at the same time, he had a 1.5 cm diameter area of hypoesthesia in the region just anterior to the tragus of his right ear. He had noted a very minor right-sided hearing loss for at least two years prior to this, but it would never last more than for a few minutes, perhaps once a month, and he never thought much about it. There was no vertigo, nausea, headaches, or other neurological symptoms associated with this.

He was 6′ 3′′ tall and weighed 180 pounds. His past medical history was pertinent for benign prostatic hypertrophy treated with finasteride since January 1998. He had no allergies to medications. He denied use of alcohol, cigarettes, or other drugs. His past surgical history was pertinent for a tonsillectomy and adenoidectomy in 1963 and an open reduction and internal fixation of a comminuted left distal radius fracture in May of 2001 with subsequent removal of hardware in August of 2001. Family history was pertinent for a sister 15 years earlier having a vestibular neuronitis versus chronic labyrinthitis secondary to aspartame.

Between January and August 5, 2002, he had five such prolonged episodes, along with the episodes that lasted for a few minutes. On August 5^th^, he woke up with one such episode that lasted 10 hours before it went away. On August 8^th^, he had another episode that started at noon and unlike his previous episodes, lasted three days with a severe tinnitus and hearing loss. He contacted an otolaryngologist and was started on prednisone. He had an audiogram on August 9^th^ that showed a right-sided high frequency sensorineural hearing loss (see table 1). By the time he saw the otolaryngologist on August 12^th^, the hearing loss resolved clinically and another audiogram was done and was much improved and similar to a previous audiogram he had done in 1987, except for a remaining 35-decibel loss at 8000 Hertz (Hz) (see table 1). At the time of his otolaryngologist appointment, his physical exam was normal. A magnetic resonance imaging scan of the brain with and without gadolinium was normal.

**Table 1:** Hearing threshold levels in dB (re: ANSI-1969) of case report 2 for each ear at different points in time

	Frequency (in Hz)	Thresholds in dB HL (re: ANSI-1969)				
		1/16/87	8/9/02	8/12/02	11/11/02	11/10/03
Right ear						
	250		0	0	5	10
	500	5	0	0	0	0
	1000	0	5	0	0	0
	2000	0	0	0	0	0
	3000	0	10			0
	4000	0	25	0	5	0
	6000	10	40	10	10	10
	8000	10	55	45	30	20
Left ear						
	250		5	5	0	5
	500	10	5	5	0	0
	1000	5	10	0	10	5
	2000	0	5	5	5	0
	3000	5				10
	4000	5	15	15	10	15
	6000	10			20	25
	8000	35	30	25	35	30

When the otolaryngologist did not have an explanation for his symptoms, the patient remembered that his sister had the adverse reaction to aspartame 15 years earlier. Up until now, he had consumed foods with aspartame, undeterred by his sister's experience. At this time, he was consuming aspartame in the form of one to two cans of Caffeine Free Diet Coke® along with a bowl of Fiberall® cereal a day. Thereafter he stopped consuming aspartame in any form. Over the next two months, he had no more severe episodes of tinnitus and hearing loss but did have two further episodes of milder right-sided tinnitus and hearing loss (30% of prior intensity) and only lasting 3-4 hours. Thinking that it might not be due to aspartame, he drank a can of Caffeine-free Diet Coke® a day for four days in a row. On each of these days, he had a mild episode of right-sided tinnitus and hearing loss for 2-3 hours each day. He permanently stopped his aspartame consumption after that. He had one milder episode of tinnitus and hearing loss a couple of weeks after he finished his challenge.

Follow-up audiograms showed a slowly improving 8000 Hz hearing loss: November of 2002 showed only a 20 decibel loss compared to 1987 and November of 2003 showed a 10 decibel loss compared to 1987 (see table 1).

Primary diagnosis is tinnitus and sensorineural healing loss secondary to aspartame.

Except for six mild reoccurrences of his symptoms within 24 hours of unintentionally consuming something with aspartame (hypoesthesia anterior to the tragus and mild tinnitus) lasting 2-4 hours, he has been symptom free for over six years now.

## Conclusion

Aspartame had a vestibulocochlear toxicity in a pair of siblings suggesting an idiosyncratic genetic predisposition to aspartame toxicity. In addition, the cochlear toxicity in case report 2 took at least 15 months to clear after his cessation of aspartame use suggesting that aspartame's cochlear toxicity can be long lasting.

Patients with dizziness, vertigo, tinnitus, and hearing loss present not only to otolaryngologists and neurologists, but also to primary care clinicians frequently. Even though the yield may be low, asking them about their aspartame consumption and suggesting cessation of its use, may prove helpful for some.

## Abbreviations

ENG: electronystagmograph; FDA: Food and Drug Administration.

## Consent

Written informed consent was obtained from the patients for publication of these case reports. A copy of the written consent is available for review by the Editor-in-Chief of this journal.

## Competing interests

The authors declare that they have no competing interests.

## Authors' contributions

DK obtained copies of her medical records when her health clinic closed down in the late 1980s and contributed both subjective and objective findings to case report 1. PP contributed to case report 2 and did the literature search. All authors read and approved the final manuscript.
